# In Silico Discovery of a Novel Potential Allosteric PI3Kα Inhibitor Incorporating 3-(2-Chloro-5-fluorophenyl)isoindolin-1-one to Target Head and Neck Squamous Cell Carcinoma

**DOI:** 10.3390/biology14070896

**Published:** 2025-07-21

**Authors:** Wenqing Jia, Xianchao Cheng

**Affiliations:** 1 School of Chemistry and Chemical Engineering, Qilu Normal University, Jinan 250200, China; 2 Tianjin Key Laboratory on Technologies Enabling Development of Clinical Therapeutics and Diagnostics (Theranostics), School of Pharmacy, Tianjin Medical University, Tianjin 300070, China

**Keywords:** allosteric PI3Kα inhibitors, RLY-2608, head and neck squamous cell carcinoma, scaffold hopping

## Abstract

Phosphatidylinositol 3-kinase alpha (PI3Kα) is frequently mutated in head and neck squamous cell carcinoma (HNSCC), leading to the constitutive activation of the PI3K/Akt pathway, which promotes tumor cell proliferation, survival, and metastasis. PI3Kα allosteric inhibitors demonstrate therapeutic potential as both monotherapy and combination therapy, particularly in patients with *PIK3CA* mutations or resistance to immunotherapy, through the precise targeting of mutant PI3Kα. Compared to ATP-competitive PI3Kα inhibitors such as Alpelisib, the allosteric inhibitor **RLY-2608** exhibits enhanced selectivity for mutant PI3Kα while minimizing the inhibition of wild-type PI3Kα, thereby reducing side effects such as hyperglycemia. To date, no allosteric PI3Kα inhibitors have been approved for clinical use. To develop novel PI3Kα inhibitors with improved safety and efficacy, we employed a scaffold hopping approach to structurally modify **RLY-2608** and constructed a compound library. Based on the structural information of the PI3Kα allosteric site, we conducted the systematic virtual screening of 11,550 molecules from databases to identify lead compounds. Through integrated approaches, including molecular docking studies, target validation, druggability evaluation, molecular dynamics simulations, and metabolic pathway and metabolite analyses, we successfully identified a promising novel allosteric PI3Kα inhibitor, **H-18** (3-(2-chloro-5-fluorophenyl)isoindolin-1-one). **H-18** has not been previously reported as a PI3Kα inhibitor, and provides an excellent foundation for subsequent lead optimization, offering a significant starting point for the development of more potent PI3Kα allosteric inhibitors.

## Simple Summary

*PIK3CA* mutations lead to abnormal activation of phosphatidylinositol 3-kinase alpha (PI3Kα), promoting the development of head and neck squamous cell carcinoma (HNSCC). Compared with traditional ATP-competitive PI3Kα inhibitors, such as Alpelisib, the allosteric inhibitor **RLY-2608** has strong selectivity for mutant PI3Kα and does not cause the side effect of hyperglycemia (the main reason for the limited clinical application of ATP-competitive PI3Kα inhibitors). The development of novel allosteric PI3Kα inhibitors can significantly benefit patients with *PIK3CA* mutations. We used the scaffold hopping method to modify the structure of **RLY-2608** to discover novel PI3Kα inhibitors with diverse structures and high selectivity. The results showed that the docking score between **H-18** (35.9705 kcal/mol) and PI3Kα was higher than that between **RLY-2608** (21.4709 kcal/mol) and PI3Kα, and that **H-18** selectively targeted PI3Kα by interacting with key amino acids at the allosteric site. **H-18** exhibited good safety characteristics and excellent pharmacokinetic properties. This study advances the development of PI3Kα allosteric inhibitors and provides new ideas for overcoming HNSCC.

## 1. Introduction

Head and neck cancer (HNC) is a malignant tumor of the upper respiratory and digestive tracts. According to global cancer statistics, HNC is the sixth most common cancer worldwide, with over 900,000 new cases reported annually. Both its incidence and mortality rates have shown a continuous upward trend over the past decade [1,2,3]. From a pathological perspective, more than 90% of HNC cases in the clinical setting are HNSCC, which is the most prevalent subtype. These tumors originate from malignant transformations of squamous epithelial cells in areas such as the oral mucosa, oropharynx, and larynx and are characterized by strong invasiveness and a high rate of early lymph node metastasis [4,5,6].

Molecular pathology research has shown that dysregulated activation of the PI3Kα pathway plays a key role in HNSCC progression, primarily driven by gain-of-function mutations in *PIK3CA*. The hotspot mutations of this gene are primarily distributed across three key sites: the E542 and E545 sites in the helical domain of exon 9 (mutated to lysine, E542K/E545K), and the H1047 site in the kinase domain of exon 20 (mutated to arginine or leucine, H1047R/L) [7,8,9]. Among these, the E542K/E545K mutation disrupts the electrostatic interaction between the p110α subunit and the nSH2 domain of the p85α regulatory subunit, releasing the autoinhibitory effect and leading to the constitutive activation of PI3Kα. The H1047R mutation significantly enhances its affinity for the cell membrane and catalytic activity by altering the conformation of the kinase domain. Notably, mutations in p85α (encoded by PIK3R1) are relatively rare in HNSCC, but a truncated mutant, P65α (lacking the C-terminal region after position 571), can persistently activate the PI3K pathway by abnormally enhancing p110α membrane localization [10]. From a protein structural perspective, approximately 80% of *PIK3CA* oncogenic mutations are concentrated in four functional regions: ABD, C2, helical, and kinase domain. Genomic analysis of 530 HNSCC patients in the TCGA database revealed that 97 cases (18.3%) harbored *PIK3CA* mutations, with the E542K, E545K, and H1047R/L variants accounting for 90% of the total mutations, highlighting their critical role in HNSCC pathogenesis [11,12,13].

**Figure 1:**
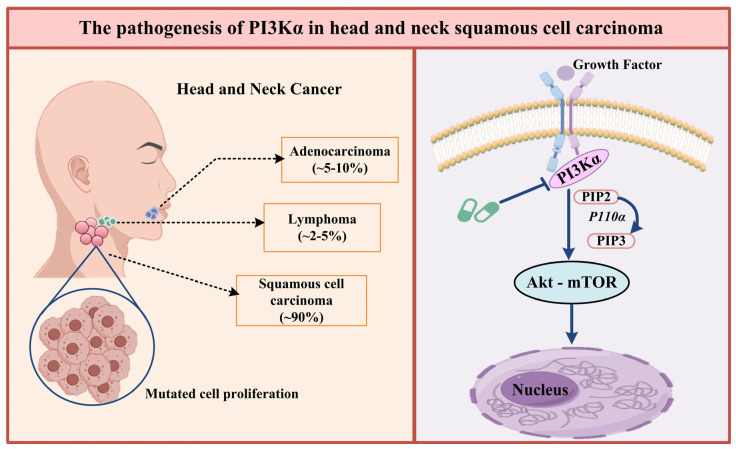
The pathogenesis of PI3Kα, inhibition by PI3Kα inhibitors, and its relationship with HNSCC.

As a classic pathway in tumor signal transduction research, PI3Kα/Akt/mTOR plays a key regulatory role in carcinogenesis. Activation of this pathway begins with PI3Kα, which catalyzes the conversion of phosphatidylinositol-4,5-bisphosphate (PIP2) into the second messenger phosphatidylinositol-3,4,5-trisphosphate (PIP3). PIP3 binds to the pH domain of Akt, mediates its translocation to the cell membrane, and induces conformational changes. This process promotes the phosphorylation of Akt at Thr308 by PDK1 and Ser473 by the mTORC2 complex, ultimately leading to Akt activation. Once activated, Akt modulates downstream signaling cascades, thereby influencing critical cellular processes, including tumor cell proliferation and survival. This pathway has been confirmed to be closely associated with malignant phenotypes such as tumor cell proliferation, metabolic reprogramming, and resistance to apoptosis (Figure 1) [14,15,16].

**Figure 2:**
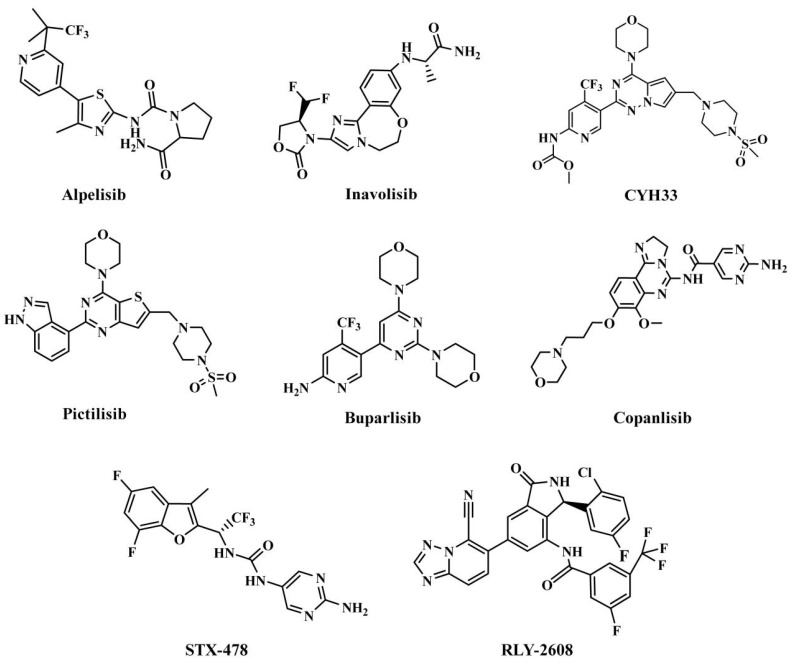
Reported PI3Kα inhibitors against *PIK3CA* mutant HNSCC.

The treatment strategies for HNSCC using PI3Kα inhibitors are mainly divided into two categories: ATP competitive PI3Kα inhibitors and allosteric PI3Kα inhibitors. Multiple ATP competitive PI3Kα inhibitors have been discovered. Alpelisib (Figure 2), an oral selective PI3Kα inhibitor, has been approved for the treatment of breast cancer harboring *PIK3CA* mutations. A phase II clinical trial for HNSCC (NCT04632992) is currently underway, focusing on evaluating its early anti-tumor activity and safety in HNSCC with abnormal activation of the PI3K/Akt/mTOR pathway [17]. Inavolisib (Figure 2) combines PI3Kα inhibition with mutant degradation, demonstrating nanomolar-level inhibitory efficacy and high selectivity in an in vitro models [18]. A phase I study (NCT06496568) is currently exploring the feasibility of monotherapy for advanced solid tumors with *PIK3CA* mutations, including recurrent/metastatic HNSCC. However, ATP-competitive inhibitors face clinical limitations, with approximately 50% of patients experiencing hyperglycemia as a side effect, necessitating frequent dose adjustments. Additionally, the acquired resistance observed during Alpelisib treatment (secondary *PIK3CA* mutations or bypass signaling activation) leads to disease progression in approximately 30% of patients, significantly limiting their clinical utility [18]. CYH33 (Figure 2) is a PI3Kα inhibitor that demonstrates an objective response rate (ORR) of 14.3% in the treatment of solid tumors harboring *PIK3CA* mutations, including HNSCC (NCT03544905) [19]. In addition, pictilisib, buparlisib, and copanlisib effectively inhibited the activity of PI3Kα and exerted anti-HNSCC effects (Figure 2) [20,21,22].

Currently, three allosteric PI3Kα inhibitors have been developed: **RLY-2608**, STX-478, and LOXO-783 (Figure 2) [23]. **RLY-2608** is an orally allosteric mutant-selective PI3Kα inhibitor with anti-tumor activity. In *PIK3CA*-mutant xenograft models, **RLY-2608** demonstrated significant antitumor efficacy with negligible effects on insulin levels, indicating minimal disruption of glucose homeostasis [24]. **RLY-2608** elicited objective tumor responses in two patients diagnosed with advanced hormone receptor-positive breast cancer with kinase or helical domain *PIK3CA* mutations, with no observed WT PI3Kα-related toxicities. The first human trial assessing **RLY-2608**, a mutant-selective PI3Kα inhibitor, was conducted as monotherapy in patients with advanced solid tumor and in combination with fulvestrant for advanced breast cancer [NCT05216432] [24]. STX-478 is an oral allosteric inhibitor developed by Scorpion Therapeutics, with 14-fold selectivity towards the H1047R mutant compared to the wild type. Preclinical studies have shown that it can induce sustained tumor regression in *PIK3CA* mutant tumor xenograft models without causing metabolic disorders [25]. LOXO-783 is an allosteric inhibitor designed for *PIK3CA* hotspot mutations and its efficacy in solid tumors, including HNSCC, is currently being evaluated in phase I/II studies (NCT05307705). Preclinical data show that it has nanomolar inhibitory activity against E542K/E545K/H1047R PI3Kα [26].

Allosteric inhibitors selectively target mutation-induced allosteric pockets, and their mode of action has three advantages: (1) avoiding interactions with highly conserved sequences in the ATP-binding region and significantly reducing off-target effects; (2) maintaining the physiological function of wild-type PI3Kα and reducing metabolic toxicity; (3) maintaining inhibitory activity against acquired drug resistance mutations [25,26,27]. These features make it possible to overcome the limitations of ATP competitive inhibitors, thereby providing a new direction for the precise treatment of *PIK3CA*-mutant HNSCC.

Currently, the primary targeted drugs for treating HNSCC are ATP competitive PI3Kα inhibitors, which often cause severe metabolic toxicity owing to insufficient target selectivity. In contrast, allosteric PI3Kα inhibitors can address these issues. However, the development of allosteric inhibitors targeting *PIK3CA*-mutated PI3Kα is still in its early stages. Emerging allosteric inhibitors demonstrating selective binding and inhibition of mutant PI3Kα variants (including H1047R) are presently undergoing initial clinical evaluations. This highlights the urgent need to develop novel allosteric inhibitors. **RLY-2608** represents a novel class of pan-mutant-selective PI3Kα inhibitors, demonstrating potent inhibition against both kinase domain (H1047R) and helical domain (E545K) activating mutations while maintaining 7–10-fold selectivity over wild-type PI3Kα [28]. To develop novel PI3Kα inhibitors with improved safety and efficacy, this study optimized **RLY-2608’s** structure using a scaffold hopping approach, guided by its binding mechanism with the PI3Kα allosteric pocket. Using computer-aided drug design techniques, structurally novel allosteric PI3Kα inhibitors have been discovered. This work reports the successful identification of low-toxicity, highly selective PI3Kα allosteric inhibitors through scaffold hopping, molecular docking studies, target validation, druggability evaluation, molecular dynamics simulations, and metabolic pathway and metabolite analyses.

## 2. Materials and Methods

### 2.1. Correlation Analysis Between Genes and HNSCC

To investigate gene-HNSCC correlations, the expression of *PIK3CA* in pan-cancer was analyzed using TIMER 2.0 (http://timer.cistrome.org/, accessed on 28 April 2024). We chose cancer exploration on the homepage of TIMER 2.0 and searched using *PIK3CA* as the keyword to analyze its expression in different tumors and normal tissues. The results were presented in a visual format. The COSMIC database (https://cancer.sanger.ac.uk/cosmic, accessed on 30 April 2024) was searched using *PIK3CA* as the keyword to analyze the mutation status and main mutation forms of genes in PI3Kα. *PIK3CA* was searched as a keyword in the Human Protein Atlas database (https://www.proteinatlas.org/, accessed on 3 May 2024), tissue was selected, and the expression of *PIK3CA* in different organs was analyzed.

### 2.2. Scaffold Hopping of ***RLY-2608***

Scaffold hopping achieves a balance between activity retention and molecular novelty through structural innovation, serving as a key strategy in drug discovery to break through the “me-too” trap and enable “first-in-class” development. When combined with computational tools, this can significantly enhance the success rate of drug design. Andreas Varkaris et al. elucidated the binding mode of the **RLY-2608** (R configuration)-PI3Kα allosteric pocket and the mechanism of **RLY-2608** targeting active sites is as follows: (1) the aminoisoxindole core can from effective space filling of the allosteric pocket, including a more extensive interaction with Y1021, along with a stronger hydrogen bond to the backbone of D1018; (2) the phenyl group with 5-fluoro substitution in **RLY-2608** allows for better filling of hydrophobic pockets; (3) the 2-chloro substitution introduces a halogen bond with the backbone carbonyl of E1012 [24]. In summary, on the basis of occupying the allosteric pocket, this type of inhibitor also needs to interact with the key amino acids Y1021, D1018, and E1012 in the binding site, enabling it to precisely target PI3Kα.

To develop novel PI3Kα inhibitors with improved safety and efficacy, this study optimized **RLY-2608’s** structure using a scaffold hopping approach, guided by its binding mechanism with the PI3Kα allosteric pocket. The **RLY-2608** structure was optimized by scaffold hopping in Discovery Studio 3.5 (Accelrys, San Diego, CA, USA). The process of scaffold hopping was as follows: (1) Building a high-quality fragment database. (2) Selecting modification points. (3) Searching the fragment database to obtain compound. Discovery Studio 3.5 was employed to perform scaffold hopping for optimization of **RLY-2608’s** structure via a three-step process: (1) the creation of a robust fragment database, (2) the selection of optimal modification points, and (3) database screening to yield modified compounds. When carrying out structural modifications on **RLY-2608**, the R/S configurations were taken into account.

### 2.3. In Silico Screening Employing Molecular Docking Techniques

Virtual screening has been widely employed in drug discovery. Virtual screening via molecular docking was performed using Discovery Studio 3.5 (Accelrys, San Diego, CA, USA). The allosteric binding site of PI3Kα (PDB ID: 8TSD, https://www.rcsb.org/, accessed on 20 May 2024) was defined with the “Define and Edit Binding Site” tool. A binding pocket (radius: 10.3 Å; center coordinates: X = 15.6, Y = −14.89, Z = −32.86) was constructed around key residues (GLN809, LEU812, THR813, ILE910, LEU911, PHE937, LEU938, LYS941, VAL952, PHE1002, MET1010, GLU1012, LEU1013, ASP1018, TYR1021, ILE1022) using the “From Current Selection” module. The screening model was constructed based on the binding mode of RLY-2608 (R configuration) to the allosteric site. We used the “Prepare Ligands” in Small Molecules of Discovery studio 3.5 to process the ligands. The ligands’ conformations were generated by the procedures of generating possible states by ionization at target pH 7.0 ± 2.0, desalting, retaining chiralities from the 3D structure, and geometry minimization with the CHARMm forcefield. Then, all the small molecules were screened by the CDOCKER module.

### 2.4. Compounds’ Target Prediction

SuperPred 3.0 (Last update: March 2022, https://prediction.charite.de/index.php, accessed on 26 August 2024) is an online web server analysis tool that can be used to predict target information for small-molecule drugs, and plays an important role in drug discovery and the identification of similar drugs [29]. Target predictions made by SuperPred rely on 2D and 3D structural similarity analyses of compounds. The query compound was matched against over 341,000 compounds and 1800 targets in the database, which contained 665,000 compound–target interaction records. Here, we input small-molecule structures into the target prediction module of SuperPred to predict the potential molecular targets.

### 2.5. The Evaluation of Druggability

As outlined in this section, the structural characteristics and pharmacokinetic properties were assessed using ADMET lab 3.0 (https://admetmesh.scbdd.com/, accessed on 3 September 2024) and SwissADME (http://www.swissadme.ch/, accessed on 20 September 2024) computational platforms. The evaluation included key physicochemical and pharmacokinetic parameters, including molecular weight (MW), lipophilicity (LogP), rotatable bonds (nRot), hydrogen bond acceptors (nHA), hydrogen bond donors (nHD), topological polar surface area (TPSA), plasma clearance rate (CL_plasma_), half-life (T_1/2_), bioavailability score, skin permeability (Log *K*p), and volume distribution (VDss). Toxicity assessments for compounds, including LD_50_, cardiotoxicity, cytotoxicity, and mutagenicity, were conducted using the ProTox-3.0 prediction platform (https://tox.charite.de/protox3/, accessed on 29 September 2024). The drug-likeness criteria for allosteric PI3Kα inhibitors are shown in Table S1.

### 2.6. Molecular Dynamics Simulations

Molecular dynamics (MD) simulations were performed using GROMACS 2020.3 software. The amber99sb-ildn force field and the general Amber force field (GAFF) were used to generate the parameters and topologies of proteins and ligands, respectively. For specific methods, please refer to our previously published article [30].

### 2.7. Analysis of Metabolic Pathways and Metabolites

The metabolic pathways and metabolites of **H-18** were analyzed using BioTransformer 3.0 (https://biotransformer.ca/new, accessed on 6 February 2025) to comprehensively investigate the properties of this compound. Specifically, the selected metabolic pathways included phase I (CYP450) and phase II transformation. Subsequently, the SDF file of **H-18** was uploaded, and the submit button was clicked. Finally, the data presented were organized and analyzed.

## 3. Results

### 3.1. PIK3CA Is Highly Correlated with HNSCC

**Figure 3:**
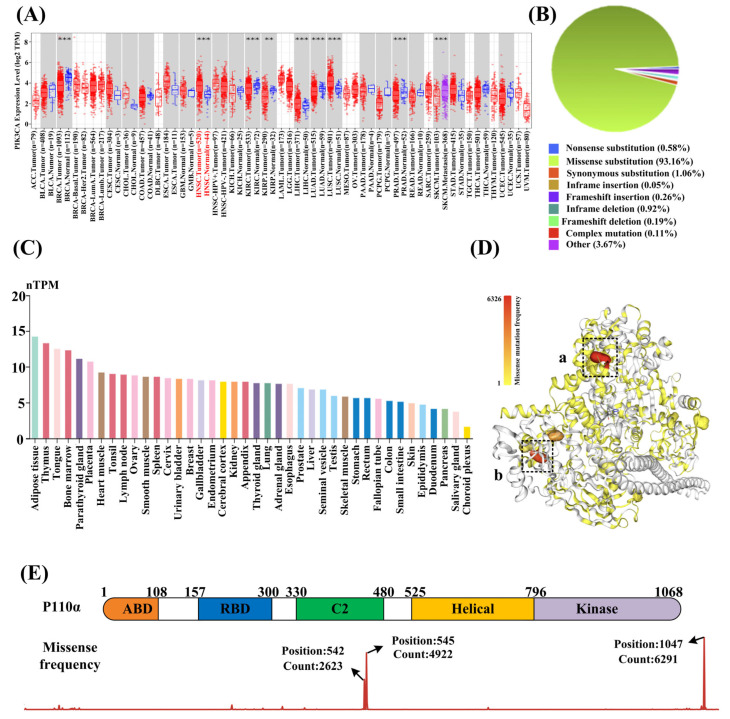
(**A**) This visualization compares *PIK3CA* expression between tumor (red) and normal (blue) tissues across different cancers by TIMER 2.0 database. The cancer types are listed along the bottom axis, while expression levels are shown on the vertical axis. For cancer types with a normal control group, the background color is displayed in gray. HNSCC is labeled in red. * *p* < 0.05, ** *p* < 0.01, *** *p* < 0.001. (**B**) An overview of the types of mutation according to the COSMIC database. (**C**) The expression of *PIK3CA* in different types of organ according to the Human Protein Atlas database. The X-axis represents the organ type, and the Y-axis represents the *PIK3CA* expression level. (**D**) Mutation sites in PI3Kα. The redder the color, the higher the mutation frequency according to the COSMIC database. The dotted boxes represent the region with a relatively high mutation frequency. Among them, “a” represents the kinase region and “b” represents the helix region. (**E**) Structure composition, hotspot mutation sites, and main mutation types of p110α according to the COSMIC database.

Using the TIMER 2.0 database, we conducted pan-cancer analysis of *PIK3CA* expression. The results demonstrated widespread *PIK3CA* expression across various tumor types (Figure 3A). Transcripts Per Million (TPM) values were used to compare the gene expression levels between cancerous and adjacent normal tissues. From Figure 3A, it could be seen that the expression levels of *PIK3CA* in HNSCC, BRCA, KIRC, LIHC, LUAD, LUSC, PRAD, and SKCM were significantly higher than that in normal tissues (*p* < 0.001). Figure 3B shows that the types of mutations in PI3Kα protein were mainly missense mutations (93.16%). The expression of *PIK3CA* in different organ types was analyzed using the Human Protein Atlas database. *PIK3CA* was highly expressed in adipose tissue, thymus, and tongue, with tongue cancer being the major tumor form of HNSCC (Figure 3C). Next, based on the COSMIC database, the mutation sites in the PI3Kα protein were analyzed. Figure 3D shows that there were two regions with high mutation frequencies in the kinase (a) and helical (b) regions. The amino acids with higher mutation frequency were H1047 (kinase region), E545 (helical region), and E542 (helical region). Among them, histidine (H) at position 1047 was most likely to mutate into arginine (R), and glutamic acid (E) at positions 545 and 542 was most likely to mutate into lysine (K) (Figure 3E). Collectively, these analyses demonstrated a strong association between HNSCC and *PIK3CA*, where mutational events resulted in PI3Kα hyperactivation. This compelling evidence underscores the therapeutic potential of PI3Kα inhibitors for HNSCC treatment.

### 3.2. Scaffold Hopping of ***RLY-2608***

**Figure 4:**
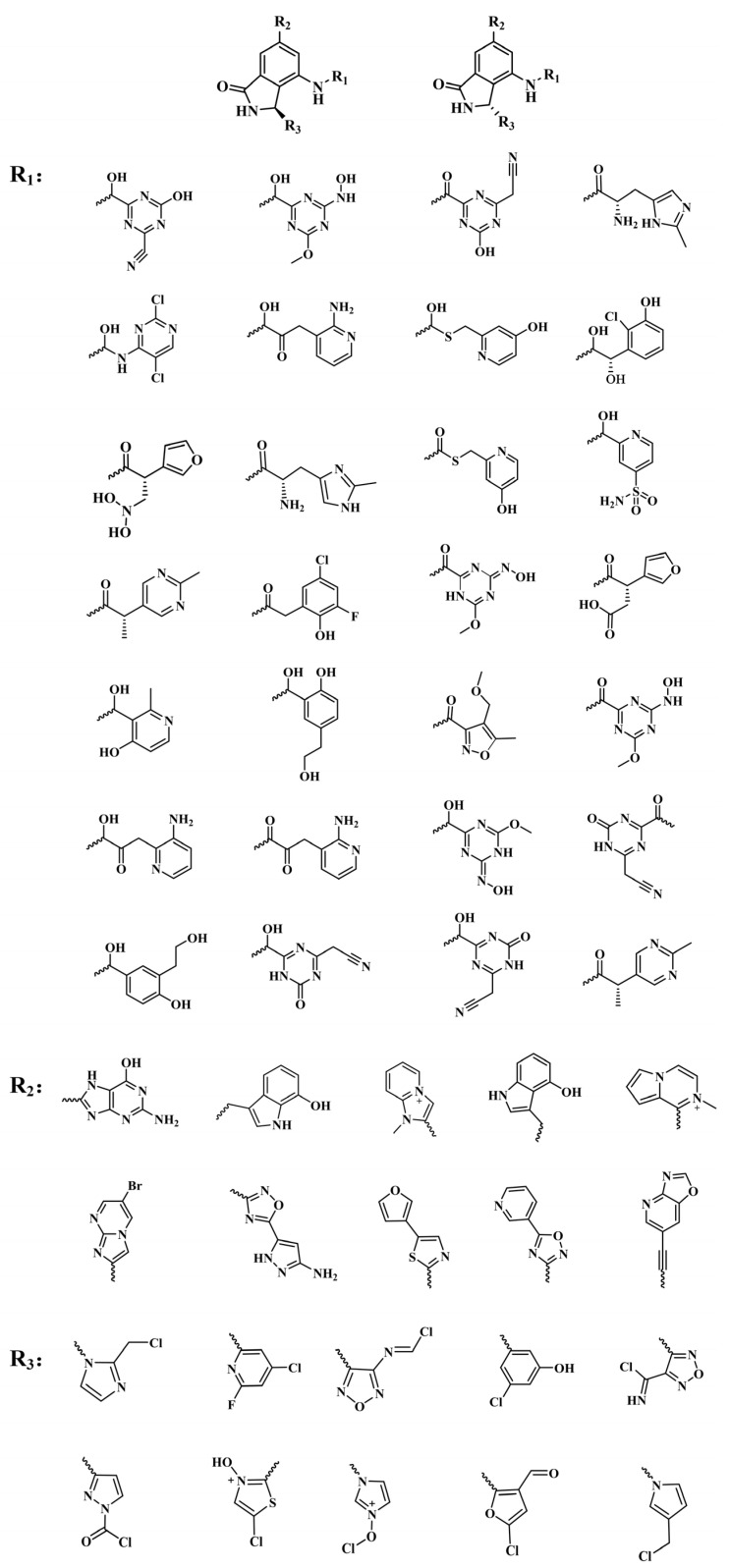
Partial fragments of R_1_, R_2_, and R_3_ in structural optimization of **RLY-2608**.

**RLY-2608** is an orally available, allosteric PI3Kα inhibitor demonstrating selective activity against mutant variants. In *PIK3CA*-mutant xenografts, it significantly suppressed tumor growth while maintaining glucose homeostasis. In **RLY-2608**, the aminoisoxindole core can from effective the space filling of the allosteric pocket and generate a stronger H-bond to the backbone of D1018. In addition, the 2-chloro substitution introduces a halogen bond with the backbone carbonyl of E1012. Generally, various interactions, such as H-bonds, hydrophobic effects, and electrostatic forces, significantly contribute to the stability of receptor–ligand complexes. Among these, H-bonds play a crucial role in determining binding affinity and stabilizing conformations [24]. Therefore, the design of novel PI3Kα inhibitors capable of stably binding to allosteric sites should focus on forming additional hydrogen bonds with these pockets while maintaining favorable safety and metabolic stability profiles. Here, using aminoisoxindole as the core structure, modifications were made to R_1_, R_2_, and R_3_ to obtain structurally diverse compounds targeting PI3Kα, all of which have not been previously reported. Through the structural optimization of **RLY-2608**, 11,550 novel compounds were generated for further investigation (Figure 4).

### 3.3. In Silico Screening Employing Molecular Docking Techniques

We performed the structural optimization of **RLY-2608** to create 11,550 derivatives. Subsequent cdocker simulations were used to analyze the compound–PI3Kα interactions and identify nine compounds with enhanced binding. The chemical structures and -cdocker energy values of the selected compounds are listed in Table 1.

**Figure 5:**
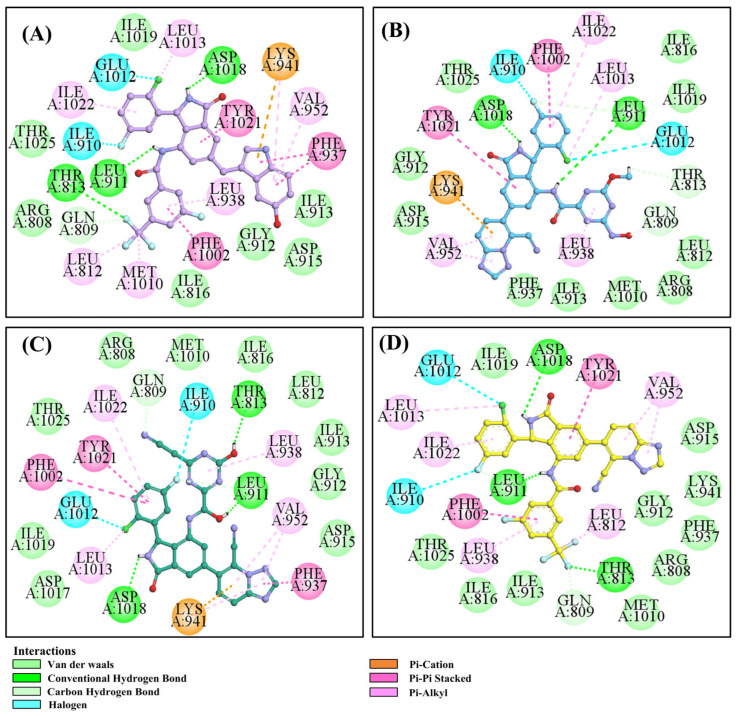
Two-dimensional (2D) interaction diagrams of **H-18** (**A**), H-72 (**B**), H-872 (**C**) and **RLY-2608** (**D**) bound to the allosteric pocket of PI3Kα.

Virtual screening revealed that the nine compounds exhibited higher -cdocker energies than **RLY-2608**. Notably, **H-18**, **H-72**, and **H-872** displayed -cdocker energies of 35.9705, 35.5813, and 35.4137 kcal/mol, respectively, surpassing that of **RLY-2608** (21.4709 kcal/mol). Figure 5A–D illustrate the binding modes of **H-18**, **H-72, H-872**, and **RLY-2608** at allosteric sites. As shown, all compounds formed H-bond interactions with ASP1018 and LEU911. Additionally, **H-18** and **H-872** formed pi–pi interactions with TYR1021, PHE937, and PHE1002, respectively; **H-72** established pi–pi interactions with TYR1021 and PHE1002. The chlorine atoms in **H-18**, **H-72, H-872**, and **RLY-2608** could all form halogen bond interactions with GLU1012 at the allosteric site.

As is well known, chirality is closely related to the activity of compounds. Figure S1 shows the interaction between **H-413** (enantiomer of **H-18**) and the active site. According to Figure 5A and Figure S1, they could all form identical H-bonds with the key amino acid residue ASP1018, and also with the residues (LEU911 and GLN809) in the PI3Kα allosteric pocket. The significant difference was that **H-18** allowed chlorine atoms to form a halogen bond with GLU1012, which was a key amino acid for compounds targeting the allosteric pocket. Meanwhile, the -cdocker energy of **H-18** (35.9705 kcal/mol) was higher than that of compound **H-413** (31.2859 kcal/mol). All evidence suggests that the S configuration of **H-18** is superior to the R configuration of H-413.

To better observe the binding mode of **H-18** with PI3Kα, we analyzed its 3D binding mode. As can be observed from Figure S2, **H-18** could bind to the allosteric site of PI3Kα just like **RLY-2608**, and its position in the active center highly overlapped that of **RLY-2608**. Therefore, it is very likely that **H-18** can stably bind to the target, just like **RLY-2608**. Full-length sequence modeling was performed using AlphaFold3 and **H-18** and **RLY-2608** were docked with the full-length sequence protein. The binding pocket was basically consistent with the active pocket of the previously resolved crystal protein (Figure S3).

### 3.4. The SuperPred Web Server Was Utilized to Predict Targets of the Top 9 Compounds

**Figure 6:**
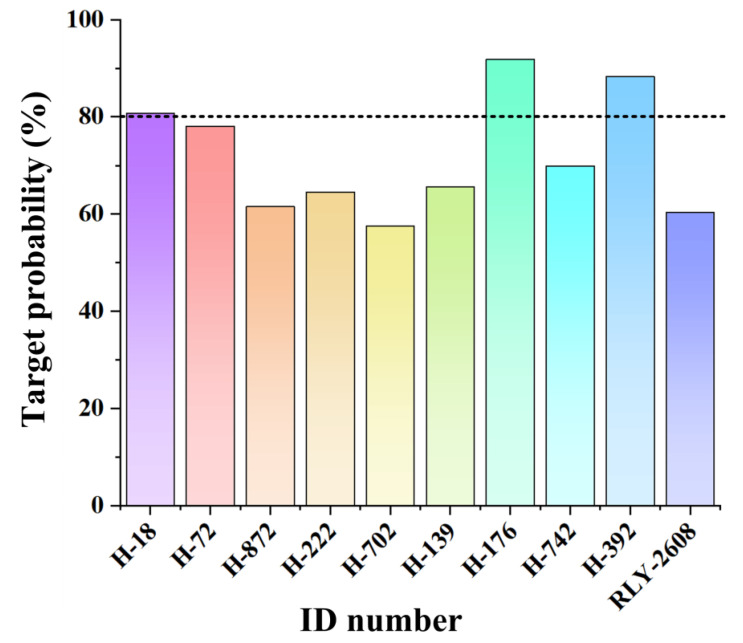
The prediction of target probability of the compounds against PI3Kα.

Based on prediction analysis, PI3Kα emerged as a high-probability target. Multiple compounds exhibited stronger binding affinities to PI3Kα than **RLY-2608** (60.31%), as shown in Figure 6 and Table 2. Notably, three candidate compounds demonstrated high targeting probabilities, exceeding 80%: **H-18** (80.72%), **H-176** (91.76%), and **H-392** (88.32%).

### 3.5. The Evaluation of Druggability

In this study, we evaluated the structural characteristics of the screened molecules. As illustrated in Table S2, **H-18**, **H-139**, and **H-392** exhibited superior properties compared with **H-72**, **H-872**, **H-222**, **H-702**, **H-176**, and **H-742** in terms of MW, LogP, nRot, nHA, nHD, and TPSA.

Compounds with LD_50_ values above 2000 mg/kg are considered minimally toxic, whereas those below 500 mg/kg pose significant toxicity risks. **H-18** exhibited low acute toxicity with an estimated LD_50_ of 2000 mg/kg, demonstrating favorable safety characteristics compared with **RLY-2608** (300 mg/kg, Table 3). **H-18** demonstrated the highest LD_50_ value among all the screened compounds (Table 3). Therefore, we selected **H-18** for further evaluation.

Drug clearance was assessed using CL_plasma_, where values below 5 mL/min/kg indicated low clearance. Both **H-18** (0.863 mL/min/kg) and **RLY-2608** (1.049 mL/min/kg) were within this range (Table 4). The T_1/2_ values of **H-18** and **RLY-2608** were 1.438 and 1.697 h, respectively, indicating that they were short-half-life drugs (Table 4). Additionally, bioavailability scores for both compounds were 0.17 (Table 4). The Log *K*p values of **H-18** and **RLY-2608** were −5.27 cm/s and −6.10 cm/s, respectively (Table 4). The VDss values of **H-18** and **RLY-2608** were 3.608 L/kg and 1.792 L/kg, respectively (Table 4). The synthetic accessibilities of **H-18** and **RLY-2608** were 4.22 and 4.27, respectively (Table 4).

In the toxicity assessment, the cardiotoxicity, mutagenicity, and cytotoxicity of **H-18** were predicted to be inactive with probabilities of 81%, 66%, and 69%, respectively (Table 5).

### 3.6. Molecular Dynamics Simulation of ***H-18***/***RLY-2608***-PI3Kα System

**Figure 7:**
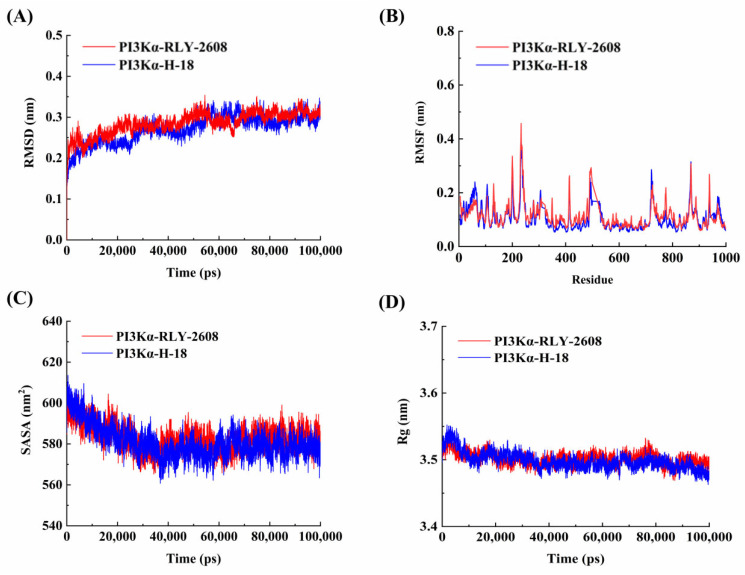
The results analysis of PI3Kα–ligand complexes throughout 100 ns molecular dynamics simulations. (**A**) The RMSD of protein–ligand complexes; (**B**) the RMSF of protein–ligand complexes; (**C**) the SASA during the simulations; (**D**) the Rg during the simulations.

The root mean square deviation (RMSD) is a key metric for assessing structural stability in molecular simulations, as it quantifies the positional variation of specific atoms relative to a reference structure. When the RMSD stabilizes, it indicates that the system has equilibrated [31]. As depicted in Figure 7A, **RLY-2608**-PI3Kα and **H-18**-PI3Kα reached equilibrium after 30 ns, demonstrating the reliability of the simulation. The RMSD values were 0.298 ± 0.017 nm for **RLY-2608**-PI3Kα and 0.288 ± 0.019 nm for **H-18**-PI3Kα, with the latter exhibiting the highest stability.

The root mean square fluctuation (RMSF) quantifies the deviation of individual atoms from their mean positions over time, reflecting the local flexibility of different protein regions [32]. As shown in Figure 7B, the RMSF values for **RLY-2608**-PI3Kα and **H-18**-PI3Kα were 0.114 ± 0.049 nm and 0.107 ± 0.053 nm, respectively. The lowest RMSF observed in the **H-18**-PI3Kα system suggests that **H-18** enhances the structural stability of PI3Kα compared to that of **RLY-2608**-PI3Kα.

The solvent-accessible surface area (SASA) measures the extent of protein surface exposure to solvent molecules, with lower values typically indicating greater system stability [33]. As illustrated in Figure 7C, the SASA values of PI3Kα in both **RLY-2608** and **H-18**-PI3Kα complexes exhibited a consistent downward trend throughout the simulation. The average SASA values were 580.964 ± 4.904 nm^2^ for the **RLY-2608-**PI3Kα system and 577.656 ± 4.721 nm^2^ for the **H-18**-PI3Kα system, respectively. Notably, the **H-18**-PI3Kα complex demonstrated the lowest SASA, suggesting enhanced stability compared to **RLY-2608**-PI3Kα, which aligns with the RMSF results.

The radius of gyration (Rg) serves as a measure of protein structural compactness. The smaller the Rg value, the more compact the protein structure [34]. As shown in Figure 7D, the **H-18**-PI3Kα (3.493 ± 0.007 nm) exhibited greater structural compactness than the RLY-2608-PI3Kα (3.499 ± 0.008 nm). This reduced Rg, coupled with the previously observed lower RMSF and SASA values, consistently indicates that the **H-18**-PI3Kα complex maintained enhanced stability throughout the simulation period.

**Figure 8:**
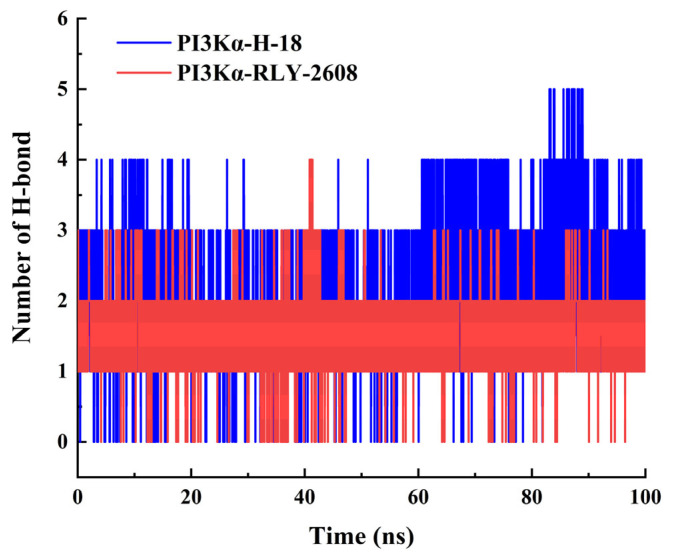
The curve of the number of H-bonds during 100 ns simulations.

To characterize the protein–ligand interactions, we performed H-bond analysis during the simulation trajectory. Following system equilibration, the average number of H-bonds formed was 2.185 ± 0.801 for the **H-18**-PI3Kα complex and 1.813 ± 0.476 for **RLY-2608**-PI3Kα (Figure 8), confirming stable H-bond interactions in both systems. The number of H-bonds in **H-18**-PI3Kα is higher than that in **RLY-2608**-PI3Kα.

During the equilibrium phase, we calculated the binding energies of the **H-18**-PI3Kα and **RLY-2608**-PI3Kα complexes using the MMPBSA method. The total binding energy was divided into four components: electrostatic interactions, van der Waals forces, and polar/nonpolar solvation effects (Table 6). The **H-18**-PI3Kα complex demonstrated stronger binding (−111.699 kJ/mol) than **RLY-2608**-PI3Kα (−65.091 kJ/mol) and the main interaction energies were electrostatic and van der Waals interactions.

**Figure 9:**
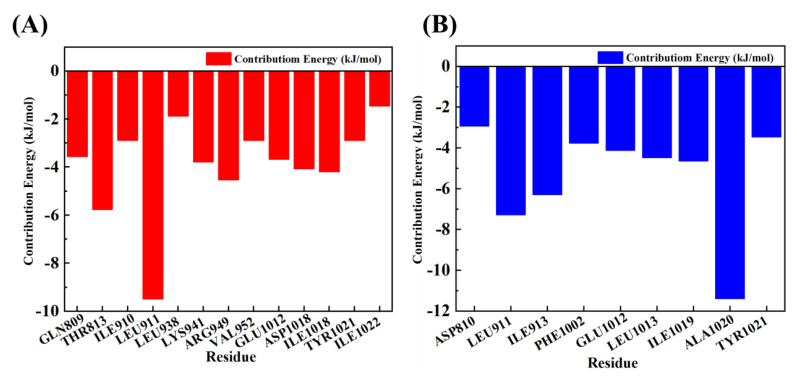
(**A**) Interaction energy decomposition for PI3Kα-**RLY-2608** complex. (**B**) Interaction energy decomposition for PI3Kα-**H**-**18** complex.

Energy decomposition analysis was conducted to quantify the contribution of individual residues to the allosteric PI3Kα–ligand interactions. The results demonstrated that **RLY-2608** formed critical contacts with GLN809, THR813, LEU911, LYS941, ARG949, GLU1012, ASP1018, and TYR1021 (Figure 9A), and **H-18** formed critical contacts with LEU911, ILE913, PHE1002, GLU1012, LEU1013, ILE1019, ALA1020, and TYR1021 (Figure 9B).

**Figure 10:**
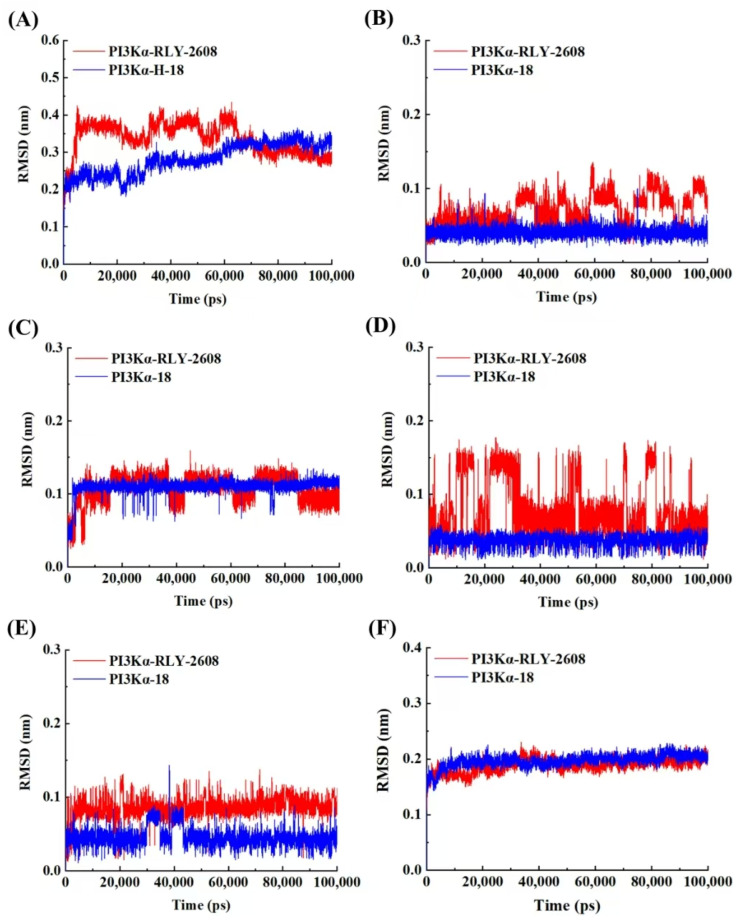
(**A**) The RMSD of the A-loop. (**B**) The RMSD of DFG. (**C**) The RMSD of the HRD motif. (**D**) The RMSD of the gatekeeper residue. (**E**) The RMSD of the catalytic lysine. (**F**) The RMSD of the α-C Helix.

By comparing the RMSD of six regions, namely, A-loop (Figure 10A), DFG (Figure 10B), HRD motif (Figure 10C), gatekeeper residue (Figure 10D), catalytic lysine (Figure 10E), and α-C Helix upon drug binding (Figure 10F), it was found that there was no significant difference in the RMSD between the **RLY-2608** and **H-18** systems in the A-loop and α-C Helix upon reaching the drug binding regions. However, in the DFG, HRD motif, gatekeeper residue, and catalytic lysine regions, the **RLY-2608** system showed greater fluctuations, indicating that **H-18** binds to the protein more stably and has a better inhibitory effect.

### 3.7. Analysis of Metabolic Pathways and Metabolites of ***H-18*** and ***RLY-2608***

**Figure 11:**
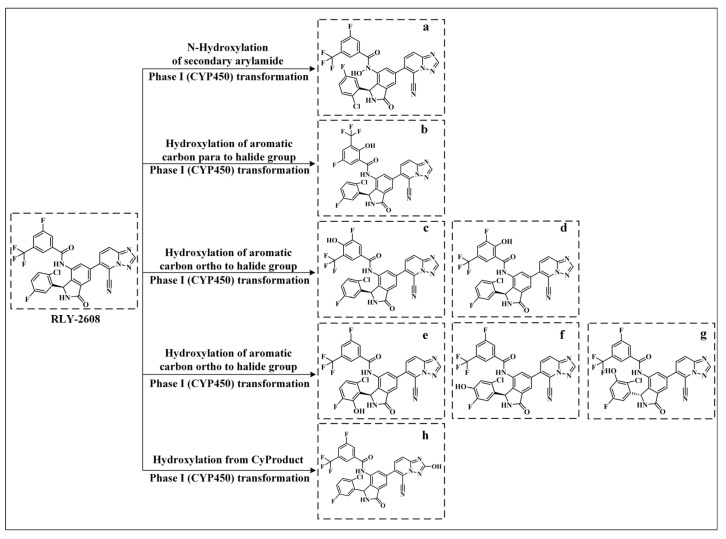
Metabolites and metabolic pathways of **RLY-2608**. a–h represent metabolites.

**RLY-2608** is an allosteric inhibitor that achieves the highly selective inhibition of PI3Kα mutants by binding to the allosteric site of the PI3Kα. To date, there have been no reports on the metabolic pathways or metabolites of **RLY-2608**. In this study, we employed the BioTransformer 3.0 platform to investigate its potential metabolites for the first time. As shown in Figure 11, **RLY-2608** primarily undergoes phase Ⅰ (CYP450) transformation in the liver. The N-hydroxylation of the secondary arylamide refers to the CYP450-mediated hydroxylation of the nitrogen atom in a secondary arylamide (Ar-NH-CO-R), yielding an N-hydroxyarylamide (Ar-N(OH)-CO-R). In **RLY-2608**, the nitrogen atom of the secondary arylamide was hydroxylated via this metabolic pathway to generate metabolite a. The hydroxylation of the aromatic carbon para to the halide group describes the hydroxylation of the carbon atom at the para position relative to a halogen (e.g., Cl, Br, F) on an aromatic ring. This common phase I oxidation reaction may enhance compound polarity or provide a site for subsequent phase II conjugation (e.g., glucuronidation). In **RLY-2608**, the hydrogen on the carbon para to the fluorine atom of the amide-linked benzene ring was hydroxylated to form metabolite b. The hydroxylation of the aromatic carbon ortho to the halide group involves the hydroxylation of the carbon atom adjacent to a halogen (ortho position) on an aromatic ring. In **RLY-2608**, the hydrogens on the carbons ortho to the fluorine atom of the amide-linked benzene ring were hydroxylated to produce metabolites c and d. For the benzene ring connected to the isoindolin-1-one moiety, which bears fluorine and chlorine atoms, the hydrogens on the carbons ortho to these halogens were hydroxylated to generate metabolites e, f, and g. Hydroxylation from CyProduct typically refers to hydroxylation mediated by CYP450-generated hydroxylated products (CyProduct). The hydrogen atom bonded to the carbon of the triazole group underwent this metabolic pathway to yield metabolite h.

**Figure 12:**
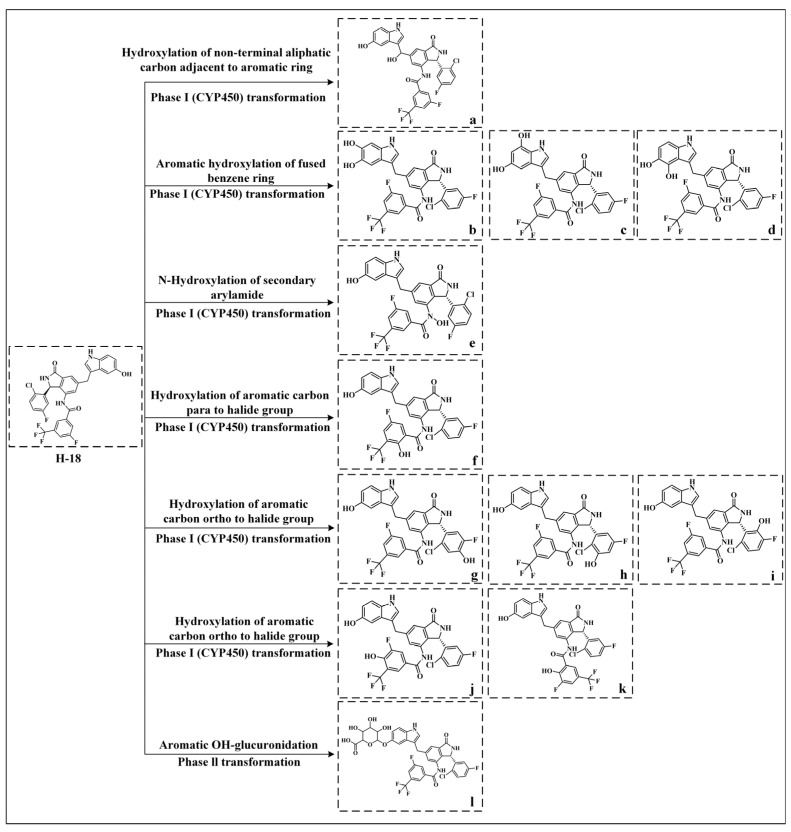
Metabolites and metabolic pathways of **H-18**. a–l represent metabolites.

The metabolic processes and metabolites of **H-18** are shown in Figure 12. In phase I metabolism, the hydroxylation of a non-terminal aliphatic carbon adjacent to an aromatic ring refers to a typical oxidative reaction targeting the non-terminal carbon atoms (i.e., the internal methylene or methine groups) in aliphatic chains directly connected to aromatic rings. In **H-18**, the hydrogen atom on the methylene group attached to the benzene ring underwent hydroxylation via this metabolic pathway to generate metabolite a. The aromatic hydroxylation of the fused benzene ring refers to the CYP450-mediated direct oxidation of C-H bonds to C-OH bonds in polycyclic aromatic hydrocarbons (PAHs) or drug molecules containing fused ring systems, representing a common oxidative pathway in drug metabolism. In **H-18**, the hydrogen atoms on the benzene ring of the benzopyrrole moiety underwent this metabolic pathway to generate metabolites b, c, and d. Additionally, the nitrogen atom in the secondary amide group of **H-18** was hydroxylated via the N-hydroxylation reaction of the secondary arylamide to yield metabolite e, while the hydrogen atom para to the fluorine substituent on the amide-linked benzene ring was hydroxylated through a hydroxylation reaction of the aromatic carbon para to the halide group to form metabolite f. Furthermore, the hydrogens on carbons ortho to both fluorine and chlorine atoms on the isoindolin-1-one-connected benzene ring were hydroxylated via the hydroxylation reaction of the aromatic carbon ortho to the halide group, producing metabolites g, h, and i, with the hydrogens adjacent to the fluorine atom on the amide-linked benzene ring similarly hydroxylated to generate metabolites j and k. In phase II metabolism, aromatic OH-glucuronidation describes the conjugation of drugs or metabolites containing phenolic hydroxyl groups with uridine diphosphate glucuronic acid (UDPGA), catalyzed by UDP-glucuronosyltransferases (UGTs) to form glucuronides, a core phase II reaction that enhances drug polarity and facilitates excretion. The hydroxyl group attached to the benzene ring of the indole moiety in **H-18** could participate in this metabolic pathway to produce metabolite I.

## 4. Conclusions

*PIK3CA* is closely associated with HNSCC. Its activating mutations (such as E542K, E545K, and H1047R) persistently activate the PI3K/Akt/mTOR signaling pathway, promoting tumor cell proliferation, survival, and metastasis [35]. In HNSCC, *PIK3CA* exhibits a high mutation rate (particularly in HPV-negative subtypes) [36]. Mutations in this gene not only enhance tumor invasiveness but may also modulate the immune therapy response by altering the tumor microenvironment, thereby establishing PI3Kα as both a critical biomarker and therapeutic target for HNSCC targeted therapies.

PI3Kα inhibitors have achieved remarkable progress in the targeted therapy of *PIK3CA*-mutated HNSCC. By selectively inhibiting the PI3Kα to block the PI3K/Akt/mTOR pathway, these inhibitors significantly prolong patients’ progression-free survival [37]. Allosteric PI3Kα inhibitors target the non-ATP binding sites of PI3Kα, demonstrating enhanced isoform selectivity and reduced off-target toxicity [38]. This novel class of inhibitors overcomes the drug resistance and metabolic side effects (such as hyperglycemia) associated with conventional ATP competitive inhibitors. In *PIK3CA*-mutated HNSCC, these allosteric inhibitors provide sustained suppression of oncogenic signaling pathways while minimizing interference with normal PI3Kβ/δ/γ functions in healthy tissues, thereby improving the therapeutic window. Currently, allosteric inhibitors, including **RLY-2608**, show promise as safer precision therapy drugs for patients with HNSCC [39].

By modifying the structure of **RLY-2608**, we found that **H-18** can stably bind to PI3Kα. **H-18** (a 3-(2-chloro-5-fluorophenyl)isoindolin-1-one derivative) exhibits rational structural design and superior properties. Isoindoline forms an H-bond with the key residue ASP1018 while establishing π-π stacking with TYR1021. Notably, the chlorine atom participated in halogen bonding with GLU1012. Furthermore, the amino group attached to the isoindoline scaffold formed an additional H-bond with LEU911, and the fluorine atom interacted with THR813 and GLN809 through H-bonds. The newly incorporated 1H-indol-5-ol moiety contributed to binding affinity via π-π interactions with PHE937. MD was used to evaluate the stability of the **H-18**-PI3Kα complex. These comprehensive interactions enabled **H-18** to achieve stable binding with PI3Kα, significantly enhancing its inhibitory potency. The results of the ADMET study indicated that **H-18** has excellent drug-likeness. Its parameters such as CL_plasma_, T_1/2_, and VDss met the requirements for druggability. During the hepatic metabolism process, **H-18** underwent phase I metabolism such as oxidative reactions and the phase II metabolism of aromatic OH-glucuronidation, which enhanced the polarity of the compound and promoted drug excretion. In addition, the safety of **H-18** has been significantly improved. The LD_50_ of **H-18** was 2000 mg/kg, which was much higher than that of **RLY-2608** (300 mg/kg), and the probabilities of cardiotoxicity and cytotoxicity were very low. This may be related to the introduction of 3-methyl-1H-indol-5-ol. **H-18** is a potential allosteric inhibitor that can also avoid the common hyperglycemia-related side effects of ATP-competitive inhibitors. SciFinder analysis confirmed the structural novelty of **H-18** and highlighted its significance in further development.

Scaffold hopping and virtual screening techniques have become core tools in modern drug design, especially in the development of kinase inhibitors. These technologies are efficient, low-cost, and can quickly discover kinase inhibitors with novel structures and strong targeting effects. The results indicate that **H-18** can stably bind to allosteric sites and has the potential to inhibit PI3Kα activity.

However, this technology has certain limitations and cannot fully reflect the real biological environment. Therefore, it is necessary to further validate the results through in vitro and in vivo experiments. For example, a kinase assay can be used to study the inhibitory effect of **H-18** on PI3Kα, the MTT assay can be used to detect the effect of **H-18** on HNSCC cell proliferation inhibition, and flow cytometry can be used to explore the effect of **H-18** on HNSCC cell cycle arrest and apoptosis regulation. In the future, we will reveal the anti-HNSCC effect and mechanism of **H-18** through in vitro and in vivo studies, and analyze the three-dimensional protein structures through MD, X-ray crystallography, and nuclear magnetic resonance (NMR), laying a preclinical foundation for the development of **H-18**.

## Figures and Tables

**Table 1:** The structures of compounds and docking results.

ID Number	Chemical Structure	Molecular Formula	-Cdocker Energy (kcal/mol)
**H-18**	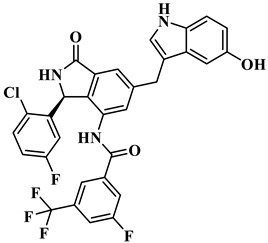	C_31_H_19_ClF_5_N_3_O_3_	35.9705
**H-72**	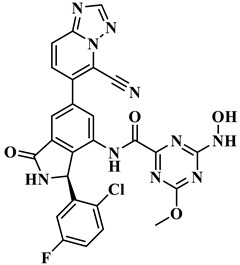	C_26_H_16_ClFN_10_O_4_	35.5813
**H-872**	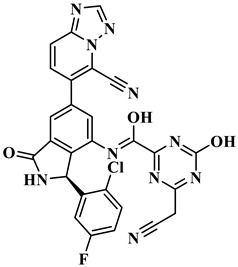	C_27_H_14_ClFN_10_O_3_	35.4137
**H-222**	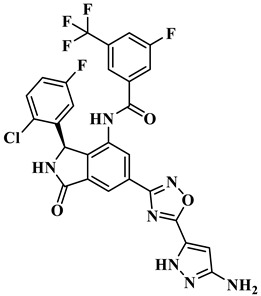	C_27_H_15_ClF_5_N_7_O_3_	34.0786
**H-702**	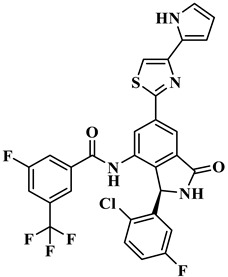	C_29_H_16_ClF_5_N_4_O_2_S	32.8416
**H-139**	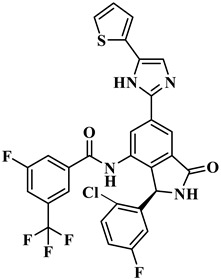	C_29_H_16_ClF_5_N_4_O_2_S	32.0775
**H-176**	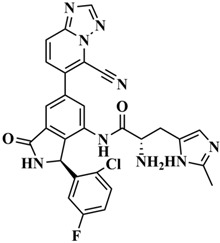	C_28_H_21_ClFN_9_O_2_	30.5798
**H-742**	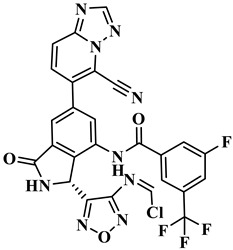	C_26_H_12_ClF_4_N_9_O_3_	30.4041
**H-392**	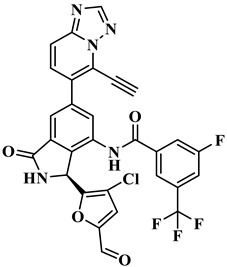	C_29_H_14_ClF_4_N_5_O_4_	27.8655
**RLY-2608**	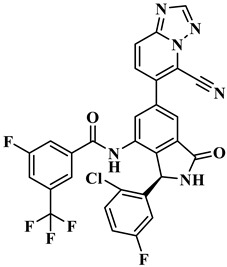	C_29_H_14_ClF_5_N_6_O_2_	21.4709

**Table 2:** The probability and model accuracy of the top 9 compounds against PI3Kα using SuperPred target prediction web server.

Target Name	Probability	Model Accuracy
**H-18**	80.72%	94.33%
**H-72**	78.09%	97.47%
**H-872**	61.53%	94.33%
**H-222**	64.53%	94.33%
**H-702**	57.49%	94.33%
**H-139**	65.56%	94.33%
**H-176**	91.76%	94.33%
**H-742**	69.89%	94.33%
**H-392**	88.32%	94.33%
**RLY-2608**	60.31%	97.47%

**Table 3:** The LD_50_ of the top 9 compounds.

	H-18	H-72	H-872	H-222	H-702	H-139	H-176	H-742	H-392	RLY-2608
LD_50_ (mg/kg)	2000	300	300	1250	300	110	1120	300	300	300

**Table 4:** The ADME properties of the **H-18** and **RLY-2608**.

	H-18	RLY-2608
Cl_plasma_ ^1^ (mL/min/kg)	0.863	1.049
T_1/2_ ^2^ (h)	1.438	1.697
Bioavailability score ^3^	0.17	0.17
Log *K*p ^4^ (cm/s)	−5.27	−6.10
VDss ^5^ (L/kg)	3.608	1.792
Synthetic accessibility	4.22	4.27

^1^ CL_plasma_: plasma clearance rate; >15 mL/min/kg: high clearance; 5–15 mL/min/kg: moderate clearance; <5 mL/min/kg: low clearance. ^2^ Ultra-short-half-life drugs: T_1/2_ < 1 h; short-half-life drugs: T_1/2_ between 1 and 4 h; intermediate short-half-life drugs: T_1/2_ between 4 and 8 h; long-half-life drugs: T_l/2_ > 8 h. ^3^ Bioavailability score: probability of F > 10% in rat. ^4^ Log *K*p: skin permeation value. ^5^ VDss: volume distribution; optimal: 0.04–20 L/kg.

**Table 5:** The toxicity assessment results of the **H-18** and **RLY-2608** given by ProTox-3.0.

	H-18	RLY-2608
Cardiotoxicity	Inact81%	Inact90%
Mutagenicity	Inact66%	Inact58%
Cytotoxicity	Inact69%	Inact78%

Inact%: the probability of predicting that the compound is non-toxic. Act%: the probability of predicting that the compound is toxic.

**Table 6:** The binding free energy (kJ/mol) of PI3Kα with **H-18**/**RLY-2608**, and its components between the receptor and ligand.

Energy	H-18	RLY-2608
Van der Waals Energy (kJ/mol)	−251.206	−180.671
Electrostatic energy (kJ/mol)	−55.374	−187.826
Polar solvation energy (kJ/mol)	197.626	310.695
Nonpolar solvation energy (kJ/mol)	−27.680	−23.788
Total binding energy (kJ/mol)	−136.634	−81.590
T∆S (kJ/mol)	24.935	16.499
Total binding free energy (kJ/mol)	−111.699	−65.091

## Data Availability

Data is provided within the manuscript or Supplementary Information files.

## Supplementary Materials

The following supporting information can be downloaded at: https://www.mdpi.com/article/10.3390/biology14070896/s1. Figure S1: (A) The structure of **H-413**; (B) The 2D interaction diagram of **H-413** bound to the allosteric pocket of PI3Kα; Figure S2: (A,B) 3D diagram of the binding of **H-18** and **RLY-2608** to the allosteric site; (C) Superimposed diagram of **H-18** and **RLY-2608** in the active site; Figure S3: (A) 2D docking diagram of **H-18** and full-length protein. (B) 2D docking diagram of **RLY-2608** and full-length protein; Table S1: The drug-likeness criteria for allosteric PI3Kα inhibitors; Table S2: The evaluation of Lipinski’s rule of five for the top 9 compounds.
