# Supplementary material for: In Silico Discovery of a Novel Potential Allosteric PI3Kα Inhibitor Incorporating 3-(2-Chloro-5-fluorophenyl)isoindolin-1-one to Target Head and Neck Squamous Cell Carcinoma
Source: Biology (Basel). 2025 Jul 21;14(7):896. doi: 10.3390/biology14070896 (PMC12292759; doi:10.3390/biology14070896)
Supplement: Supplementary file 1 [file biology-14-00896-s001.zip › Table S1.pdf]

Table S1. The drug-likeness criteria for allosteric PI3Kα inhibitors

Parameter	Minimum	Maximum
LogP	-2	5
MW	100	700
nHA	0	10
nHD	0	5
nRot	0	11
TPSA (Å^2)	0	140
