# Supplementary material for: In Silico Discovery of a Novel Potential Allosteric PI3Kα Inhibitor Incorporating 3-(2-Chloro-5-fluorophenyl)isoindolin-1-one to Target Head and Neck Squamous Cell Carcinoma
Source: Biology (Basel). 2025 Jul 21;14(7):896. doi: 10.3390/biology14070896 (PMC12292759; doi:10.3390/biology14070896)
Supplement: Supplementary file 1 [file biology-14-00896-s001.zip › Table S2.pdf]

Table S2. The evaluation of Lipinski's rule of five for the top 9 compounds.

ID number	MW[1]	LogP[2]	nRot[3]	nHA [4]	nHD[5]	TPSA[6] (Å^2)
**H-18**	611.1	5.877	7	6	4	94.22
**H-72**	586.1	2.855	7	14	4	192.34
**H-872**	580.09	2.43	5	13	3	198.36
**H-222**	615.08	4.639	7	10	5	151.82
**H-702**	614.06	6.09	7	6	3	86.88
**H-139**	614.06	5.894	7	6	3	86.88
**H-176**	569.99	1.996	7	11	5	166.88
**H-742**	609.89	3.97	7	12	2	163.46
**H-392**	607.09	4.406	7	9	2	118.6
**RLY-2608**	608.08	4.987	6	8	2	112.18

1. MW: 100~700; **2.** LogP: ≤5; **3.** nRot: 0~11; **4.** nHA: 0~10; **5.** nHD: 0~5; **6.** ≤140 Å^2.
