# Supplementary figures and images for: In Silico Discovery of a Novel Potential Allosteric PI3Kα Inhibitor Incorporating 3-(2-Chloro-5-fluorophenyl)isoindolin-1-one to Target Head and Neck Squamous Cell Carcinoma
Source: Biology (Basel). 2025 Jul 21;14(7):896. doi: 10.3390/biology14070896 (PMC12292759; doi:10.3390/biology14070896)

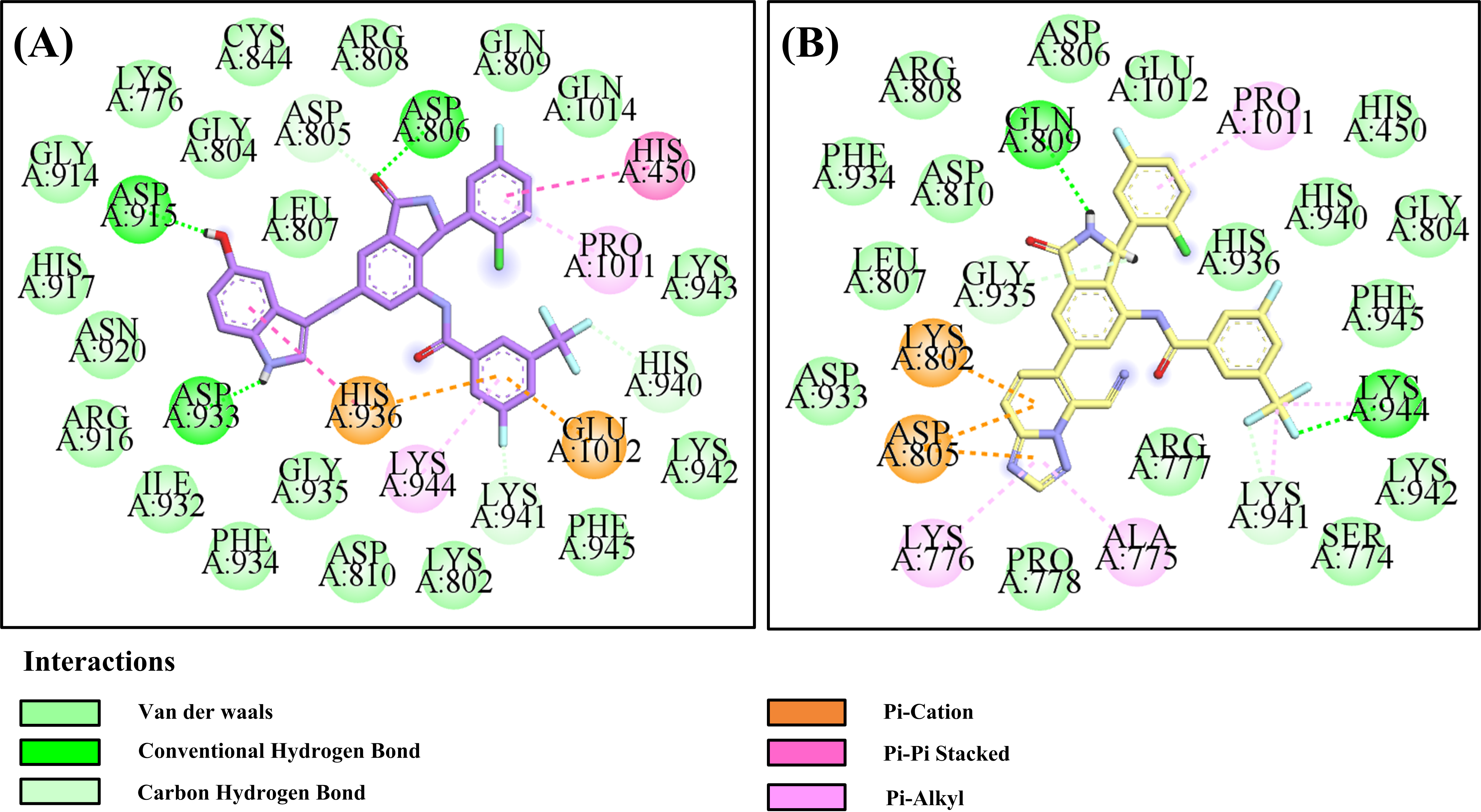

Supplement: Supplementary file 1 [file biology-14-00896-s001.zip › Figure S3.png.tif]
